# Dehydroepiandrosterone (DHEA) Serum Levels Indicate Cerebrospinal Fluid Levels of DHEA and Estradiol (E2) in Women at Term Pregnancy

**DOI:** 10.1007/s43032-021-00541-2

**Published:** 2021-03-26

**Authors:** Pardes Habib, Joseph Neulen, Shahin Habib, Benjamin Rösing

**Affiliations:** 1 Department of Neurology, Medical Faculty, RWTH Aachen University, 52074 Aachen, Germany; 2 Institute of Biochemistry and Molecular Immunology, Medical Faculty, RWTH Aachen University, 52074 Aachen, Germany; 3 Clinic for Gynecological Endocrinology and Reproductive Medicine, RWTH Aachen University, 52074 Aachen, Germany; 4 Medical Biochemistry, Department of Biochemistry, University of Leicester, Leicester, UK; 5 Medical Faculty, RWTH Aachen University, Pauwelsstrasse 30, 52074 Aachen, Germany

**Keywords:** Neuroactive steroids, Dehydroepiandrosterone, Estradiol, Progesterone, Cerebrospinal fluid, Pregnancy, Reproductive endocrinology

## Abstract

Neuroactive steroids such as dehydroepiandrosterone (DHEA), estradiol (E2), and progesterone (P4) are associated with structural and functional changes in the central nervous system (CNS). Measurement of steroid levels in the CNS compartments is restricted in accessibility. Consequently, there is only limited human data on the distributional equilibrium for steroid levels between peripheral and central compartments. While some neuroactive steroids including DHEA and E2 have been reported to convey excitatory and proconvulsant properties, the opposite was demonstrated for P4. We aimed to elucidate the correlation between peripheral and central DHEA, E2, and P4 levels in women at term pregnancy. CSF and serum samples of 27 healthy pregnant women (22–39 years) at term pregnancy were collected simultaneously under combined spinal and epidural anesthesia and used for DHEA ELISA and E2, and P4 ECLIA. All three neuroactive steroids were detected at markedly lower levels in CSF compared to their corresponding serum concentrations (decrease, mean ± SD, 97.66 ± 0.83%). We found a strong correlation for DHEA between its serum and the corresponding CSF levels (*r* = 0.65, *p* = 0.003). Serum and CSF levels of E2 (*r* = 0.31, *p* = 0.12) appeared not to correlate in the investigated cohort. DHEA serum concentration correlated significantly with E2 (*r* = 0.58, *p* = 0.0016) in CSF. In addition, a strong correlation was found between DHEA and E2, both measured in CSF (*r* = 0.65, *p* = 0.0002). Peripheral DHEA levels might serve as an indicator for central nervous levels of the neuroactive steroids DHEA and E2 in pregnant women.

## Introduction

The neuroactive steroids dehydroepiandrosterone (DHEA), estradiol (E2), and progesterone (P4) have been found to modulate neural activity to a variable degree exerting excitatory and inhibitory effects in neural tissue [1–4]. These properties are mediated by nuclear, mitochondrial, or cell membrane-bound steroid receptors, which occur in varying densities and subtypes throughout the CNS [5–7] and via nongenomic signaling pathways [8, 9].

Neuroactive steroids originate both from peripheral glands and de novo synthesis in the CNS in neurons and glial cells [10, 11]. The main serum availability of these steroids in premenopausal women derives from peripheral glands (ovaries, adrenals) [12] and in case of pregnancy from the fetoplacental unit. Extraordinarily high circulating E2 concentrations during pregnancy result from placental aromatase activity and rise by several orders of magnitude (100 to 500-fold) compared to normal cycle conditions [13].

Steroid hormones cross the blood-brain barrier (BBB) to a certain extent, but the intercompartment equilibrium between serum, cerebrospinal fluid (CSF), and neural tissue is unclear [14]. The study of intra-tissue steroid hormone formation and metabolism reveals the expression of required enzymes for steroid hormone synthesis in topical variance in the brain tissue, although there are conflicting data concerning the complete steroid biosynthetic pathways in human CNS [15]. The balance between intra-tissue de novo synthesis to systemic delivery of hormone precursors and metabolites has not yet been disentangled [16]. Thus, it is unclear how peripheral hormone changes affect central steroid distribution patterns.

Concerning the clinical impact of neuroactive steroids, there is an ongoing debate on their pathophysiology with contradictory clinical observations and research results [4]. Regular cyclic fluctuations of hormone levels are associated with CNS symptoms such as premenstrual dysphoric syndrome (PMDS) or catamenial epilepsy patterns [17]. There is no strong mechanistic basis for the assumption that estradiol is proconvulsant, nonetheless estradiol has potent neuroexcitatory effects under certain circumstances and without a doubt, estradiol influences seizure susceptibility in women with epilepsy [18–22]. In contrast, there is good evidence demonstrating anticonvulsant properties of progesterone and its metabolite allopregnanolone [23, 24].

Although the status quo of seizure frequency appears to remain controlled during pregnancy in most women with epilepsy, several studies report an increasing seizure frequency in 20–50% of these patients [25–28]. Reliable predictors of the clinical course have not yet been identified. The increase in seizure frequency is not solely due to pharmacokinetic effects or noncompliance with anti-epileptic drug (AED) medication during pregnancy. Vajda et al. found a significant rise in seizure frequency during pregnancy in an Australian registry-based case series of women with unmedicated epilepsy, excluding changes in AED availability due to altered pharmacokinetics or medication compliance in this cohort [29].

A possible cofactor modulating seizure frequency could be a pregnancy-related change toward stronger estrogen dominance in the concentration ratio of excitatory to anticonvulsant neuroactive steroids. If neuroactive steroids modulate the susceptibility for convulsive disorders in pregnancy, this would certainly also concern women at risk for eclampsia. Eclampsia refers to a new onset of tonic-clonic seizures in pregnancy and typically occurs in women with a hypertensive pregnancy disorder such as preeclampsia, HELLP (acronym for hemolysis, elevated liver enzymes, low platelets) syndrome, and gestational hypertension. Women at risk are those with pre-pregnancy overweight or obesity, pregestational diabetes, and hypertension [30, 31]. In addition, advanced maternal age, use of assisted reproductive technology, especially after frozen embryo transfer [32], and multiple pregnancy are relevant further risk factors for the occurrence of eclampsia [31].

Seizures in pregnancy are potentially life threatening for both mother and fetus. Specific diagnostic tests to guide early preventive strategies for women at risk for seizures during pregnancy are not available. Clinical data suggest that neuroactive steroids are etiologically involved in the development of seizures [18–24] and if these steroids follow specific distribution equilibria between serum and CSF, monitoring of serum hormones could reveal a prognostic endocrine risk constellation for these patients. The identification of steroid patterns in serum, reflecting CSF-concentrations of neuroactive steroids with an excitatory profile, could help to identify situations with an increased risk for seizures in pregnant women. Given the current uncertainties, an investigation of steroid fluctuation and distribution between central and peripheral compartments in clinical populations of interest would be a sensible direction for further studies.

The access to CNS tissue for steroid measurement from individuals without CNS pathology is limited for ethical reasons. Within certain diagnostic or therapeutic clinical procedures including combined spinal-epidural anesthesia, cerebrospinal fluid (CSF) is available and can be utilized for analyses. The aim of the study was to evaluate the correlations of important neuroactive steroid hormone levels between the two compartments in pregnant women.

## Subjects and Methods

This prospective study was approved by the local institutional ethics committee of the Medical Faculty, RWTH Aachen University, Aachen, Germany, in accordance to the Helsinki Declaration on Ethical Principles for Medical Research Involving Human Subjects and the Guideline for Good Clinical Practice (EK 201/14). All participants signed informed consent. A total of 27 healthy pregnant women in the 38–40th gestational week, who presented themselves for a planned cesarean section between 2014 and 2015 were included. CSF and blood samples were collected simultaneously during combined spinal-epidural anesthesia for elective cesarean section. After epidural puncture liquor was rinsed to verify correct intrathecal needle position. One milliliter of clear CSF without macroscopic blood contamination was then collected and stored in an Eppendorf tube in −80° for further analysis. Blood samples were centrifuged, and serum and CSF were assayed for DHEA, E2, and P4 concentrations in the same laboratory. To measure the DHEA concentrations in serum and CSF, an ELISA was utilized according to the instructions of the manufacturer (Enzo Life Science, Farmingdale, USA, sensitivity 2.9 pg/ml (range 12.21–50.000 pg/ml), <9% inter- and intra-assay variation). In order to measure E2 and P2 in both compartments of the patients, an electrochemiluminescence immunoassay (ECLIA, Cobas Roche Diagnostics, Mannheim, Germany) was performed according to the manufacturer’s instructions. The detection levels of ECLIA for the two hormones were as follows: E2 = LoD: 18.4 pmol/L (5 pg/ml) and LoQ: 91.8 pmol/L (25 pg/ml) and for P4 = LoD: 0.159 nmol/L (0.05 ng/ml) and LoQ: 0.636 nmol/L (0.2 ng/ml). All measured hormone levels in our cohort were within the detection level of the ELISA and ECLIA used, thus none of the patients had to be excluded from the study.

### Statistics

Data analysis and visualization were performed using GraphPad Prism (version 8.4.3, San Diego, CA, USA). Bivariate correlation of quantitative data was assessed by nonparametric Spearman’s rank correlation. A correlation coefficient of (rho = *r*) 0.00–0.19 indicates a very weak correlation, 0.20–0.39 a weak correlation, 0.40–0.59 a moderate correlation, 0.60–0.79 a strong correlation, and 0.8–1.00 a very strong correlation. A *p* value of < 0.05 was considered significant.

## Results

### Serum DHEA Levels Significantly Correlate with DHEA and E2 Levels in CSF

To elucidate a potential correlation between serum and CSF levels of the neuroactive steroids dehydroepiandrosterone (DHEA), progesterone (P4), and estradiol (E2) at term pregnancy, we included a total of 27 pregnant women (mean age: 30.5, range: 22–39 years) with elective cesarean section in the 38–40th gestational week in this study (Table 1). In all subjects, both CSF and serum samples were collected simultaneously under combined spinal and epidural anesthesia.

**Table 1:** Demographics and medical history of included women

Patients (*n*)	27
Age (years) + (range)	30.5 (22–39)
Height (m) ± SD	1.68 ± 0.06
Weight (kg) ± SD	79.4 ± 10.8
Delivery BMI (kg/m^2^) ± SD	28.3 ± 3.7
Ethnicity	C (23), As (3), Af (1)
Gestation (weeks) + (range)	38.4 (37–40)
Parity	G1–9, P0–8, A0–3
Co-morbidities	6 × hypothyroidism, 2 × gestational hypertension
Co-medications	6 × L-thyroxine (50–75), 2 × methyldopa (500–1000/d)
Indication for CS	21 × maternal request, 2 × fetal malpresentation, 3 × prior uterine surgery, 1 × abnormal placentation

*BMI*, body mass index (weight [kg]/ height [m]^2^); *CS*, cesarean section; *C*, Caucasian; *As*, Asian; *Af*, African; *G*, gravidity; *P*, parity; *A*, abortion

**Fig. 1:**
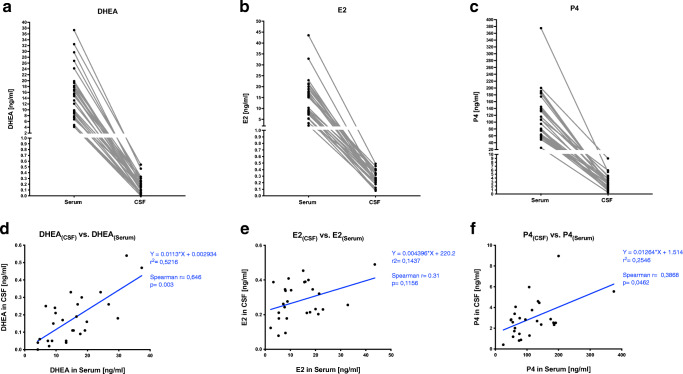
Associations between serum and CSF levels of neuroactive steroids in pregnant women. Serum and CSF of 27 women at term pregnancy was sampled during cesarean section. **a** Dehydroepiandrosterone (DHEA) concentrations, **b** estradiol (E2), and **c** progesterone (P4) in serum and CSF per subject is given, respectively. Spearman rank correlation analysis between serum and CSF revealed in **d** a significant strong correlation for DHEA (*r* = 0.65, *p* = 0.003) in **e** a non-significant correlation for E2 (*r* = 0.31, p=0.12) and in **f** a weak correlation for P4 (*r* = 0.39, *p* = 0.046)

We observed a 98.83% (SD: ± 0.80) decrease to a mean DHEA level in CSF of 0.18 ng/ml (range: 0–0.47 ng/ml) (Fig. 1a) from a mean serum DHEA level of 15.38 ng/ml (range: 4.14–37.33 ng/ml). A similar pattern of serum and CSF levels was evident for both E2 and P4. Estrogen was found at a mean serum level of 13.9 ng/mL (range: 2.2–43.6 ng/mL) with a 97.15% (SD: ± 2.2%) reduction in CSF (mean: 281.44 ng/mL, range: 123–490 ng/mL) (Fig. 1b). Also, P4 was reduced by approximately 97.01% (SD: ± 1.66) in the CSF (mean: 2.96 ng/ml, range: 0.41–5.55 ng/ml) compared to the corresponding serum levels (mean: 114.04 ng/ml, range: 25–375 ng/ml) of pregnant women (Fig. 1c). Although all three steroids displayed similar relative decreases between serum and CSF levels, a Spearman rank correlation analysis between serum and CSF revealed a significant strong correlation only for DHEA (*r* = 0.65, *p* = 0.003) (Fig. 1d). While a significant but weak correlation (*r* = 0.39, *p* = 0.046) was detected for P4, only serum and CSF levels of E2 (*r* = 0.31, *p* = 0.12) appeared not to correlate in the investigated cohort (Fig. 1e/f).

Next, we evaluated if peripheral and central DHEA levels correlated with central E2 and P4 levels and if DHEA could be a suitable indicator for E2.

**Fig. 2:**
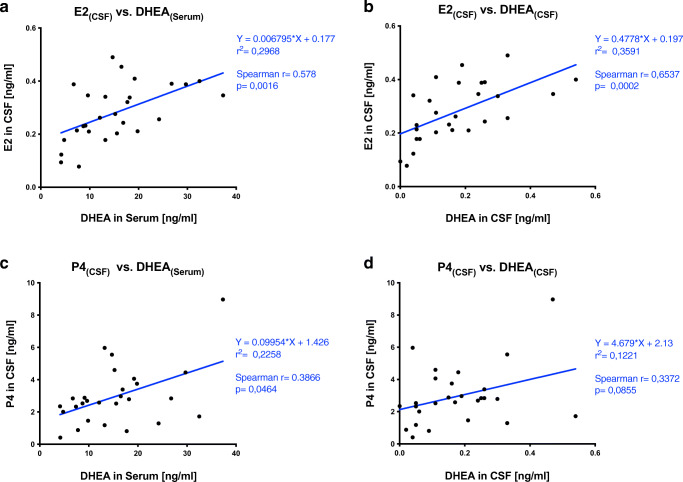
DHEA serum levels significantly correlate with CSF levels of E2 and P4 in pregnant women. DHEA levels in serum (*r* = 0.58, *p* = 0.0016) (**a**) and in CSF (*r* = 0.65, *p* = 0.0002) (**b**) display a significant correlation with central E2. DHEA levels in serum show a significant but weak correlation with P4 levels in CSF (*r* = 0.39, *p* = 0.046) (**c**), while DHEA in CSF seems not to correlate with central P4 levels (*r* = 0.34, *p* = 0.085) (**d**)

DHEA serum concentration correlated significantly with both central nervous E2 (*r* = 0.58, *p* = 0.0016) and P4 in CSF (*r* = 0.39, *p* = 0.046) (Fig. 2a/c). The correlation of peripheral DHEA levels with central E2 was stronger than with P4. While a significant and strong correlation between the steroids DHEA and E2 measured in CSF was found (*r* = 0.65, *p* = 0.0002), no significant correlation between DHEA and P4 was evident in this compartment (*r* = 0.34, *p* = 0.085) (Fig. 2b/d).

In summary, our data suggest that peripheral DHEA levels may serve as a good indicator for central nervous levels of the neuroactive steroids DHEA (*r* = 0.65, strong correlation) and E2 (*r* = 0.58, moderate correlation) in pregnant women. For P4, the intercompartment correlation was weak (*r* = 0.39) in the tested cohort.

## Discussion

The balance of neuroactive hormones is not systematically reflected or studied in relation to seizure frequency, e.g., in pregnant women with epilepsy or in eclampsia. A pregnancy is an exceptional physiological and endocrine situation with excessive hormone synthesis accompanied by highly variable serum concentrations and altered steroid metabolism compared to non-pregnant women. Alterations in blood-brain barrier function may also occur.

The correlations of serum to CSF concentrations of the measured steroid hormones (DHEA, E2, P4) vary according to the specific steroid in our cohort of 27 women at term pregnancy. Our results for pregnant women are consistent with the repeated observation in non-pregnant subjects that steroid concentrations broadly differ at least by one to two orders of magnitude between the serum to CNS compartments [33, 34]. We found the strongest inter-compartment correlation for DHEA. On the contrary, correlations for estradiol were not significant and weak but significant for progesterone. Interestingly central E2 concentration correlated best to serum DHEA (coefficient *r* = 0.58, moderate correlation) and to central DHEA (coefficient *r* = 0.65, strong correlation). We were able to find constant correlations between serum and CSF measurements, although the hormone concentrations in the serum showed a wide, pregnancy-typical dispersion.

To our knowledge this is the first study observing CSF and serum concentrations of neuroactive steroid hormones in women at term pregnancy. CSF and serum concentrations were measured from simultaneously collected samples. In line with our results, in vivo data of patients, though scarce in number, suggest that the steroidal environment in CNS versus intravascular compartments differs by a magnitude of 10 to 100 with lower concentrations in the CNS. Animal models support these findings [14, 35]. We choose a set of steroids with neuroactive potential that are most available in clinical routine analysis. DHEA is a steroidogenic precursor to E2 aromatization and passes the BBB relatively easily resulting in high correlations between blood and CSF compared to other steroids [36]. DHEAS in contrast has a higher polarity compared to DHEA. Therefore, passive BBB passage is considered limited compared to the unsulfated form [37]. Estradiol and progesterone are the two most discussed neuroactive steroids in seizure susceptibility in women with epilepsy [4].

This study reveals certain limitations. We compared CSF to serum distribution for steroid hormones with neuroactive potential in healthy pregnant women without affection of the blood-brain barrier. The measurement of neuroactive hormone concentrations in CSF is an approximation to the distribution of steroid hormones in brain tissue. Basically, topical CNS tissue concentrations may not be adequately reflected in CSF [33]. Most stable correlations were found for DHEA in CSF and temporal brain tissue [38].

Pregnancy is a distinct physiological condition that might include changes in blood-brain barrier function as well as central and peripheral steroid metabolism compared to non-pregnant subjects. Therefore, the results may not be valuable for non-pregnant women and men.

Statistical evidence base is limited due to a small sample size and observational study design. Our study population of 27 subjects was small, though comparatively large regarding published populations in this field.

ECLIA and ELISA testing were not constructed for hormone analysis in CSF [39], though immunoassays have been used for CSF measurements in many publications before. Without a doubt mass spectrometry is superior for precise steroid detection in CSF. But this technique was not available for this study. Here we wanted to show proportional hormone distributions between different compartments. Any result with immunoassay measurement in CSF definitely needs bridging studies before wider clinical applications.

Nevertheless, we think that our results contain valuable information for further research. The steroidal hormone transport across the blood-brain barrier varies according to the specific hormone [36]; however, the knowledge of precise mechanisms and distributional equilibrium between peripheral and central compartments is limited [16]. In our study we found no significant correlation for central E2 with peripheral E2 levels. This is in line with previous observations that E2 does not pass BBB well [33, 34, 36]. But we saw a moderate correlation for central E2 with peripheral DHEA levels (correlation coefficient *r* = 0.58, defined as moderate correlation). Furthermore, we could show that central DHEA correlates significantly with central E2 levels (correlation coefficient *r* = 0.65, defined as strong correlation) as with peripheral levels of DHEA (correlation coefficient *r* = 0.65, defined as strong correlation).

DHEA is the steroidogenic precursor to E2 aromatization. This is also true for CNS tissue [15]. DHEA passes the BBB relatively easily resulting in high correlations between blood and CSF compared to other steroids [36] and might be correlated with the local downstream steroid metabolism in progress. Peripheral DHEA could be a quantitatively relevant metabolic substrate for central E2 synthesis due to its BBB transport characteristics. Following this hypothesis, serum DHEA levels represent local E2 availability in the CNS more accurately than serum E2 concentrations do. The observed steroid equilibria between central and peripheral compartments lead to the hypothetical assumption that DHEA could be a suitable identifier of hormone (DHEA and E2) concentrations in CSF. However, this interpretation should be treated with caution, as the study design is not suitable for describing metabolic pathways and complete steroid biosynthesis in human CNS remains to be elucidated [15]. The variability of steroid production in pregnancy presumably encompasses different serum DHEA to P4 ratios. Assuming that increased serum DHEA concentrations indicate increased E2 levels in the CNS, an elevated serum DHEA to P4 ratio would indicate an increased E2 to P4 ratio in the central compartment. Such a shift in steroid balance would result in a stronger potential for CNS excitability due to dominating neuroactive estradiol effects over anticonvulsive progesterone effects.

The DHEA (E2) to P4 ratio would also increase with lower P4 concentrations. Interestingly, Shine et al. [40] found significantly decreased placental steroid production of E2 and P4 in women with preeclampsia, while mean DHEA levels did not differ between groups, although this finding could not be replicated by others [41].

## Conclusion

Changes in the central nervous neuroactive steroid profile toward estrogenic dominance in the course of pregnancy may be a potential modulator for an increase of convulsive events. In this regard, a peripheral indicator for the central neuroactive steroid potential would be a helpful clinical monitoring tool. From the results of our study, it can be hypothesized that the CSF estradiol concentration seems to be better indicated by the serum DEHA level than by the serum estradiol level. There was also a weak intercompartmental correlation for progesterone that reached statistical significance. This relationship and possible steroid thresholds require clinical verification studies. This could include identifying a risk population for seizures and tracking steroid concentrations and ratios in pregnancy in relation to clinical event progression.

## Data Availability

The datasets used and/or analyzed during the current study are available from the corresponding author on reasonable request.
